# Comparison of Shear Bond Strength of Resin-Modified Glass Ionomer and Composite Resin to Three Pulp Capping Agents

**DOI:** 10.5681/joddd.2013.026

**Published:** 2013-08-30

**Authors:** Amir Ahmad Ajami, Elmira Jafari Navimipour, Siavash Savadi Oskoee, Mehdi Abed Kahnamoui, Mehrdad Lotfi, Mehdi Daneshpooy

**Affiliations:** ^1^Dental and Periodontal Research Center, Tabriz University of Medical Sciences, Tabriz, Iran; ^2^Associate professor, Department of Operative Dentistry, School of Dentistry, Tabriz University of Medical Sciences, Tabriz, Iran; ^3^Associate professor, Department of Operative Dentistry, School of Dentistry, Tabriz University of Medical Sciences, Tabriz, Iran; ^4^Professor, Department of Endodontic Dentistry, School of Dentistry, Tabriz University of Medical Sciences, Tabriz, Iran; ^5^Professor, Department of Endodontic Dentistry, School of Dentistry, Tabriz University of Medical Sciences, Tabriz, Iran

**Keywords:** Resin composite, resin-modified glass ionomer, MTA, shear bond strength

## Abstract

***Background and aims.*** Present study was designed to compare the bonding strength of resin-modified glass ionomer (RMGI) and composite resin to mineral trioxide aggregate (MTA), MTA mixed with Na2HPO4 (NAMTA), and calcium-enriched mixture (CEM).

***Materials and methods.*** Thirty specimens of each CEM, NAMTA, and MTA were prepared. Composite and RMGI restorations were then placed on the samples (15 samples in six subgroups). Shear bond strength was assessed using universal testing machine. Data were analyzed with two-way ANOVA and post-hoc Tukey test. To compare the bond strength in subgroups, one-away ANOVA was applied. Significance level was set at P < 0.05.

***Results.*** Bond strength was significantly higher to composite samples compared to RMGI samples (p<0.001). The difference in bond strength of composite samples between MTA and CEM subgroups (P=0.026) as well as MTA and NAMTA subgroups (P= 0.019) was significant, but the difference between NAMTA and CEM subgroups (P=0.56) was not significant. The differences in bond strength in subgroups of RMGI group were not significant (P>0.05).

***Conclusion.*** Regarding shear bond strength to the tested substrates, composite was shown to be superior to RMGI. The bond of resin composite to MTA was weaker than that to CEM and NAMTA.

## Introduction

Direct pulp capping is defined as placing a biocompatible material on a normal pulp exposed unintentionally during removal of caries or because of a trauma to seal the pulp and prevent bacterial leakage as well as stimulating the formation of a dentin bridge to preserve pulp vitality.^1^ Materials like calcium hydroxide and mineral trioxide aggregate (MTA) have been used for this purpose. Pulp-capped teeth have to be sealed by restorative materials like amalgam or resin composite. Bond between restorative materials and pulp capping agents is very important and in the lack of a proper seal, permeation of bacteria into pulp and failure of pulp capping procedure will occur.^2^

Although MTA is a commonly used material, it has certain disadvantages such as difficult handling and reported delayed setting time of 75 minutes up to 73 hours.^3,4^ To reduce the setting time, several components have been added to MTA, including NaOCl gel and COCl_2_, but only to reduce mechanical strength of MTA. Light cured MTA has a reduced setting time but it is not as osteoconductive as conventional MTA.^5^ Another way is to use Na_2_HPO_4_ which is a buffer solution and can replace distilled water to reduce the setting time to 38.3 min, without affecting the pH.^6,7^ The x-ray diffraction (XRD) pattern also remains similar.^7^ White MTA (WMTA) mixed with Na_2_HPO_4_ (NAMTA) in comparison with conventionally mixed WMTA has been shown to be more biocompatible with no primary inflammation after implantation.^6^

On the other hand, a recently introduced material, Calcium Enriched Mixture (CEM), can be applied in direct pulp capping, apexification and root perforation treatment.^8,9^ In a case series study, CEM showed success in pulpotomy of permanent molars.^10^ CEM is an antibacterial material providing an effective seal against leakage, sets in the presence of water, and triggers the formation of hydroxyapatite and hard tissue. CEM has a desirable biocompatibility and is insoluble in water. It has been shown that its setting time is lower than 1 hour, whereas MTA needs at least 4 hours to set.^8^ Also, the high pH, antibacterial activity, ability to provide a perfect seal and biocompatibility of CEM are comparable with those of MTA.^11^ The thickness of the dentin bridge with CEM has been shown to be more than that with MTA.^12^ Manipulation of CEM is easier than MTA, does not stick to the instruments and is condensable, making CEM a preferable alternative over MTA.^8^

Glass ionomer (GI) is used as liner because of its chemical bond to dental structure and its fluoride release.^13^ Conventional and resin modified glass ionomers have been recommended as liners under resin composite restorations to reduce microleakage, a technique commonly referred to as sandwich technique.^14^

The shear bond strength of pulp capping agents to restorative materials has been an issue of concern. Shear bond strength of MTA to resin composite with two bonding systems was investigated and it was reported that the total-etch provides higher shear bond strength between MTA and composite than self-etch system.^15^ Beyrak et al showed that the Prime & Bond NT which is a total-etch adhesive produces stronger bond between MTA and compomer in comparison to three self-etch adhesion systems.^16^ It has also been demonstrated that the bond strength between CEM and composite is lower than that between resin-modified GI (RMGI) and composite.^17^ Since previous studies have not evaluated the bond strength of the newly introduced CEM and NAMTA to commonly used restorative materials, we evaluated the shear bond strength of RMGI and composite, as restorative materials and CEM and NAMTA, as substrates, considering the bond to MTA as the gold standard.

## Materials and Methods

### Preparation of samples

In this in vitro study, 90 acrylic cylindrical blocks were prepared and then in the center of the cylinders, a hole with a 4-mm diameter and a 2-mm height was created. CEM powder (Bionique Dent; Tehran, Iran) with its specific liquid as well as MTA (Dentsply; Tulsa Dental, OK, USA) and NAMTA (produced by dr. Lotfi in Tabriz Dental Faculty, Tabriz, Iran) with distilled water were mixed according to the corresponding manufactures’ instructions. The materials were placed with a carrier in prepared cavities of the specimens (30 samples each) and condensed with a condenser and flattened with spatula. The blocks were coded. Samples were kept in a humid environment with temperature of 37°C for 24 hours for the materials to set. The surfaces of the samples were sandpapered with a 600-grit sandpaper. Specimens of each material were divided into two groups (15 samples each), and received either composite resin or RMGI.

In three composite subgroups, samples were etched with 35% phosphoric acid gel (Scotchbond Etchant, 3M ESPE Dental Products, St. Paul, USA) for 15 sec, rinsed with water for 30 sec, and then dried with oil-free air syringe for 5 sec. In the next step, one-bottle adhesive (Adper TM Single Bond, 3M ESPE Dental Products, St. Paul, USA) was applied to the surface of samples with a clean microbrush (Microbrush Co., Greyton, USA). The application of adhesive was done twice and after the second one, a mild air syringe flow was applied for 2 to 5 sec to evaporate the solvent. Then the adhesive was light-cured for 15 sec (Astrulis 7, Ivaclar Vivadent, Florida, USA) adjusted on low power program with fixed intensity of 400 mW/cm^2^. Transparent plastic molds with diameter and height of 3 mm were filled with composite resin (Filtek TM Z250, A2 shade, 3M ESPE Dental Products, St. Paul, USA). Upon filling, the mold was placed on the prepared surface of the sample and the composite was condensed. Molds were light-cured for 20 sec from top and 40 sec from the lateral sides.

A similar method was used for RMGI samples. RMGI special conditioner (10% polyacrylic acid; CG Corp., Tokyo, Japan) was used for 20 sec and then rinsed for 30 sec and dried with oil-free air syringe for 5 sec. RMGI (Fuji II LC, CG Corp., Tokyo, Japan) powder was mixed with specific liquid on a glass slab with a plastic spatula, placed in translucent molds and light-cured for 40 sec in the same manner.

Samples were kept in a humid environment with temperature of 37°C for 24 hours to simulate the oral condition. Samples were then placed in universal testing machine (H5k-S, Hounsfield Test Equipment, UK) equipped with chisel like load (0.5 mm width) and tested with a velocity of 1 mm/min. The force upon sample breaking was recorded in Newton.

All samples were observed under stereomicroscope (Nikon SMZ 1000, Tokyo, Japan) with ×25 magnification to determine the type of failure as either adhesive or cohesive.

### Statistical analysis

After confirmation of normal distribution of the data with Kolmogorov Smirnov test and equality of variance between groups with Levene test, a two-way ANOVA analysis was performed with shear bond strength as dependent variable and the type of material (composite or RMGI) and type of substrate (CEM, NAMTA, or MTA) as factors. Post-hoc Tukey test was used to compare groups two by two. To investigate the difference among the subgroups, one-way ANOVA and post-hoc Tukey test were used. The significance level was set at P < 0.001.

## Results

Table 1 demonstrates mean shear bond strength of the studied samples. Two-way ANOVA showed that the difference of mean bond strengths in composite subgroups was higher than the RMGI subgroups (P < 0.001). It was shown that mean shear bond strength was significantly influenced by the type of the substrate (P < 0.001). The post-hoc Tukey test showed that the difference of shear bond strength between NAMTA and CEM is not significant (P = 0.24) but the difference of shear bond strength between CEM and MTA (P = 0.001) and between NAMTA and MTA (P = 0.001) was significant. The interaction between the type of restorative material and the type of the substrate was significant (P < 0.001).

**Table 1:** Mean shear bond strength of the studied samples in MPa

Sample	Number	Bond Strength
Composite	45	
CEM	15	18.03 ± 1.18
NAMTA	15	19.78 ± 1.56
MTA	15	12.12 ± 2.31
RMGI	45	
CEM	15	2.69 ± 0.41
NAMTA	15	3.25 ± 0.70
MTA	15	3.24 ± 0.58

**Figure 1:**
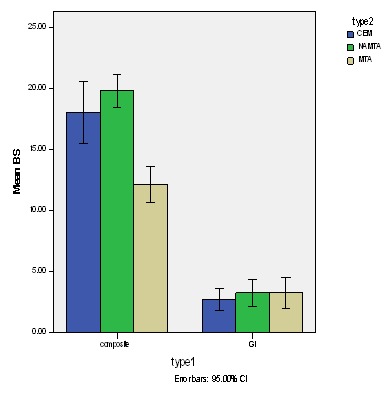
bond strength of Resin-Modified Glass Ionomer and Composite Resin in the studied samples.

Comparing the subgroups using one-way ANOVA showed that in the composite group the difference in shear bond strength between substrates was significant (P = 0.029). Two by two comparisons of subgroups with Tukey test indicated that the difference of bond strength between MTA and CEM subgroups (P = 0.026) and MTA and NAMTA subgroups (P = 0.019) was significant but the difference between CEM and NAMTA (P = 0.56) was not significant. The difference in bond strength among RMGI subgroups was not significant (P = 0.58) ( Figure 1).

## Discussion

In restorative treatments involving a pulp exposure, since clinical findings and histological events do not often coincide, prediction of severity and type of the pulpal damage is almost impossible. The clinician, however, should make every effort to protect the vitality of the pulp. Treatment modalities such as pulp capping aim to preserve pulp vitality by elimination of caries (bacteria) and using biocompatible products to provide a strong barrier against bacterial microleakage.^1^ Preserving pulpal health and sealing it during this procedure is crucial. Calcium hydroxide is the popular pulp capping agent. However, other biocompatible materials such as MTA, CEM and NAMTA have gained attention recently^1^.

After pulp capping, tooth requires a proper restoration and often composite is the first choice especially in the esthetic zone. On the other hand, in the cases in which there is not enough enamel around preparation, RMGI can be a good restorative material. In such restorations, the bond between pulp capping agents and composite resin or RMGI plays a crucial role in the sealing provided by the restoration and finally in the treatment success.^2^ Therefore, bond strengths of RMGI and composite to CEM, NAMTA and MTA surfaces were investigated in the present study.

There was a significant difference between bond strengths of the composite and RMGI groups. According to the results, the bond strengths of composite to substrates (CEM, NAMTA and MTA) were higher than those of RMGI. The higher bond strength to composite can be due to the fact that the 35% phosphoric acid removes the smear layer and provides a clean surface which creates a honey comb pattern on the samples, increasing micromechanical bond, hence high bond strength as a result of micromechanical bond to composite. The effect of acid etching on MTA properties has also been studied with scanning electron microscope (SEM) demonstrating that superficial, gel-like irregular structures and spindle-shaped crystals were removed during etching. Selective removal of matrix around crystallized structures without significant loss of cement, ends in a honeycomb structure which provides desirable surface for resin materials to bond.^18^ The bond strength, however, was seen to be lower to MTA in comparison to CEM and NAMTA in resin composite samples. Main components of MTA powder include three calcium silicate, tricalcium aluminate, tricalcium oxide and silicate oxide. NAMTA beside the mentioned components contains Na_2_HPO>_4_. CEM too contains various calcium-containing compounds such as calcium oxide, calcium carbonate, calcium phosphate, calcium silicate and synthesized calcium aluminate. These substrates are hydrophilic and set in the presence of moisture.^19^ Since there is no resin structure in CEM, NAMTA and MTA, it might be safe to say that the bond is purely micromechanical. The bond strength of MTA to composite and compomer has been studied, showing that the bond was weaker with self-etch system than with total-etch system.^15^ Reason of such difference is possibly insufficient conditioning of MTA by the self-etch adhesive system. A similar study on compomers has shown total-etch adhesive provides stronger bond strength to MTA in comparison to three self-adhesive.^16^

Considering the fact that in the composite samples, almost all failures were of cohesive type in bond substrate material, the lower bond strength to MTA in comparison to CEM and NAMTA may be attributed to the difference in cohesive strength of these substrates. This can also imply that in all three subgroup, the bond strength to composite is higher than the cohesive strengths of the substrates. In line with this observation, previous research has shown that the type of the failures of the bond in gray MTA to dentin is cohesive type and inside the MTA.^20^ However, it is interesting to note that in the latter study the cohesive strength of MTA improved over time before placement of the final restoration, leading to a lower possibility of cohesive failure.^20^

In RMGI samples, no significant difference was observed among the shear bond strengths of substrates. This can be attributed to the lower etching capability of polyacrylic acid (RMGI conditioner) in comparison with the 35% phosphoric acid (composite conditioner) with regards to the preparation of the surface and creating the honeycomb pattern. The bond between RMGI and the tested hydrophilic substrates must be mostly chemical, with micromechanical bond having a negligible effect. This hypothesis is supported by the type of the failure in experimented samples being mostly of the adhesive type (between RMGI and substrates). It can also be inferred that the bond strength between RMGI and substrate was lower than the cohesive strength.

The present study did not assess the cohesive strength of the tested materials independently, and therefore, further studies on the difference of cohesive strength between CEM, MTA and NAMTA are recommended. Precise electron microscope studies are also suggested. More studies are warranted toward the effect of different preparation methods of CEM, MTA and NAMTA on their bond strength to RMGI and composite resin.

## Conclusion

Shear bond strengths between composite and all three substrates (CEM, MTA and NAMTA) were significantly higher than those of RMGI. The bond between composite resin and MTA was lower than that between the other two substrates; however, no significant differences were observed among shear bond strengths of substrates.
